# Supplementary material for: Reconciling Mining with the Conservation of Cave Biodiversity: A Quantitative Baseline to Help Establish Conservation Priorities
Source: PLoS One. 2016 Dec 20;11(12):e0168348. doi: 10.1371/journal.pone.0168348 (PMC5173368; doi:10.1371/journal.pone.0168348)
Supplement: S1 Dataset — (ZIP) [file pone.0168348.s002.zip › Taxa/Serra Norte/SN_2013/N5S/Lista N5S-34.pdf]

CAVIDADE N5S-0034

Classe	Ordem	Fam/Outros	Gên/Outros	Espécie	S-U	U-S	Zon.
Annelida	Oligochaeta			sp.	1		E
Arachnida	Amblypygi	Charinidae	*Charinus*	jovem		X	E
Arachnida	Araneae	Araneidae		jovem		X	E
Arachnida	Araneae	Pholcidae	*Leptopholcus*	sp.1		X	E
Arachnida	Araneae	Pholcidae	*Mesabolivar*	*eberhard*	X		E
Arachnida	Araneae	Theridiidae	*Episinus*	sp.4		X	E
Arachnida	Araneae	Uloboridae	*Uloborus*	sp.1	X		E
Arachnida	Opiliones	Sclerosomatidae		jovem	X		E
Arachnida	Opiliones	Stygnidae	*Protimesius*	*longipalpis*	1		E
Entognatha	Collembola	Paronellidae		sp.1	X		E
Insecta	Coleoptera	Carabidae		sp.3	X		E
Insecta	Coleoptera	Coccinellidae		sp.1	X		E
Insecta	Coleoptera	Hydrophilidae	Hidrobiinae	sp.1		X	E
Insecta	Diptera	Cecidomyiidae	Cecidomyiinae	sp.	X		E
Insecta	Diptera	Ceratopogonidae		sp.	X		E
Insecta	Diptera	Psychodidae	*Pintomyia*	*gruta*	X		E
Insecta	Diptera	Psychodidae	*Sciopemyia*	*sordellii*		X	E
Insecta	Diptera	Tabanidae		jovem	X		E
Insecta	Homoptera	Cixiidae		sp.1		X	E
Insecta	Homoptera	Cixiidae		sp.3	X		E
Insecta	Hymenoptera	Formicidae	*Camponotus*	sp.1		X	E
Insecta	Hymenoptera	Formicidae	*Crematogaster*	sp.2	X		E
Insecta	Hymenoptera	Formicidae	*Dolichoderus*	aff. *bispinosus*	X		E
Insecta	Hymenoptera	Formicidae	*Pachycondyla*	sp.1	X		E
Insecta	Hymenoptera	Formicidae	*Pachycondyla*	*striata*		1	E
Insecta	Lepidoptera	Gelechioidea		sp.5		X	E
Insecta	Lepidoptera	Noctuoidea		sp.2	X	X	E
Insecta	Lepidoptera	Noctuoidea		sp.10		X	E
Insecta	Lepidoptera	Noctuoidea		sp.13	1		E
Insecta	Lepidoptera	Tineoidea		sp.3		X	E
Insecta	Orthoptera	Phalangopsidae	*Paraclodes*	sp.	2		E
Insecta	Orthoptera	Phalangopsidae	*Phalangopsis*	sp.		3	E
Malacostraca	Isopoda	Armadillidae		sp.1		X	E
Mammalia	Chiroptera	Emballonuridae	*Peropteryx*	sp.	2	1	E
